# Design of miRNA sponges for MDV-1 as a therapeutic strategy against lymphomas

**DOI:** 10.18632/oncotarget.23379

**Published:** 2017-12-17

**Authors:** Yuan Fang, Yuqi Zhou, Yun Zhang, Liangliang He, Chunyi Xue, Yongchang Cao

**Affiliations:** ^1^ State Key Laboratory of Biocontrol, School of Life Sciences, Sun Yat-sen University, Guangzhou 510006, China

**Keywords:** miRNA sponge, marek’s disease virus 1, meq miRNA cluster, MSB-1 cell, tumorigenicity

## Abstract

Lymphomas are solid-type tumors containing lymphoid cells. Some of latent herpesvirus infections established in B and/or T-lymphocytes could result in the formation of lymphomas. Marek's disease virus serotype 1 (MDV-1) is an avian herpes virus causing to lymphoproliferative tumors in birds, known as Marek’s disease (MD). MD has often been used as an ideal biological model for studying the pathogenesis of lymphoma diseases caused by viruses. Therefore, we used it as a research subject to study the effect of miRNA sponges on its tumorigenicity, and to develop the theoretical basis for a new anti-tumor small molecule. The miRNA sponges designed in this study specifically bind to and degrade the miRNAs of meq gene cluster of MDV-1, including miR-M2-3p, miR-M3-5p, miR-M5-3p, miR-M9-5p and miR-M12-3p.qPCR results showed that the knockdown efficiency was 85.03%, 74.97%, 47.06%, 75.33% and 62.55%, respectively. EDU staining and CCK-8 results showed that miRNA sponges inhibited the proliferation of MDV-1 transformed MSB-1 cells *in vitro*, and the proliferation rate of miRNA sponges-treated cells was about 50% of the control group. DAPI staining and Annxin V-FITC/PI double staining showed that miRNA sponges induced apoptosis in MSB-1 cells, and the apoptotic rate was increased by about 27.87% compared with the control group. The results of transwell showed that miRNA sponges could inhibit the invasion of MSB-1 cells *in vitro*, and the inhibitory rate was about 64.52%. The soft agar assay showed that miRNA sponges could inhibit the tumorigenic ability of MSB-1 cells *in vitro*, and the inhibitory rate was about 66.44%.The 60-days animal study showed that miRNA sponges could alleviate the growth inhibition of MSB-1 cells (about 14.78%) and reduce the mortality (about 16.00%). In addition, the tumor formation rate was 0 (8–12% in the control group).This study suggests that miRNA sponges can serve as an effective anti-tumor small molecule for the tumors caused by herpesvirus, with potential clinical implications.

## INTRODUCTION

Approximately 12% of global human cancers are reportedly caused by viral infections [1]. Among the few viruses that have been shown to naturally cause cancer in humans, only three of them have been shown to directly cause lymphomas, i.e. Epstein-Barr virus (EBV; human herpesvirus 4), Kaposi sarcoma-associated herpesvirus (KSHV; human herpesvirus 8), and human T-cell lymphotropic virus 1 [2–4].

Marek’s disease (MD) is an avian solid-type lymphoproliferative tumor mainly caused by Marek’s disease virus serotype 1 (MDV-1). This pathogen can rapidly develop lymphoma in a variety of visceral tissues and cause cell infiltration in peripheral nerves, leading to limb (wing) paralysis [5]. MD is a worldwide problem that inflicts an annual economic loss of about $2 billions to the chicken industry [6]. Many experiments have shown that MDV can be used as an ideal biological model for studying the pathogenesis of lymphoma disease and human cancers caused by viruses [7]. Furthermore, MD is the only viral tumor that can be prevented by vaccines. Therefore, MD received worldwide attention from medical community and virologists [8, 9].

RNA interference induced by microRNAs (miRNAs) is a powerful process that blocks gene expression in mammalian cells by triggering sequence-specific degradation of mRNAs. MiRNAs are widely involved in a variety of physiological and pathologic processes, including development, differentiation, proliferation, apoptosis, and immune activation. MiRNAs are a useful tool for viruses to reshape gene expression profiles of host cells, thereby creating a favorable microenvironment for viral replication within host cells and promoting carcinogenesis [10–13]. Twenty-six of MDV1-encoded miRNAs have been identified so far and the miRNAs of Meq cluster (MDV1-miR-M2, -miR-M3, -miR-M4, -miR-M5, -miR-M9 and -miR-M12) has been shown playing critical roles in latent infection and oncogenesis [14–16].

In the year 2007, Phillip Sharp and his colleagues have developed an efficient method for long-term suppression of miRNA function. This technique is called “miRNA sponges”, which directly “adsorb” miRNAs so that miRNA molecules cannot further bind to their native mRNA targets [17]. The miRNA sponge technology provides a timely and valuable approach that can significantly improve our understanding of miRNA function, and will aid clinical treatments for various diseases caused by dysregulation of miRNAs. However, it is still unclear whether miRNA sponges can be used as a novel anti-cancer molecule for suppressing viral miRNAs-induced tumors [18, 19].

In this study, we have designed the miRNA sponges that are specific to the miRNAs of Meq cluster. The *in vitro* studies showed that the designed miRNA sponges specifically bind to target miRNA molecules within host cells, and caused loss function of pathogenic MDV-1 miRNAs. Animal studies confirmed the effective roles of miRNA sponge in interfering with pathogenicity and tumorigenicity of MDV-1.

## RESULTS

### Design and construct Meq-cluster specific miRNA sponges

**Figure 1:**
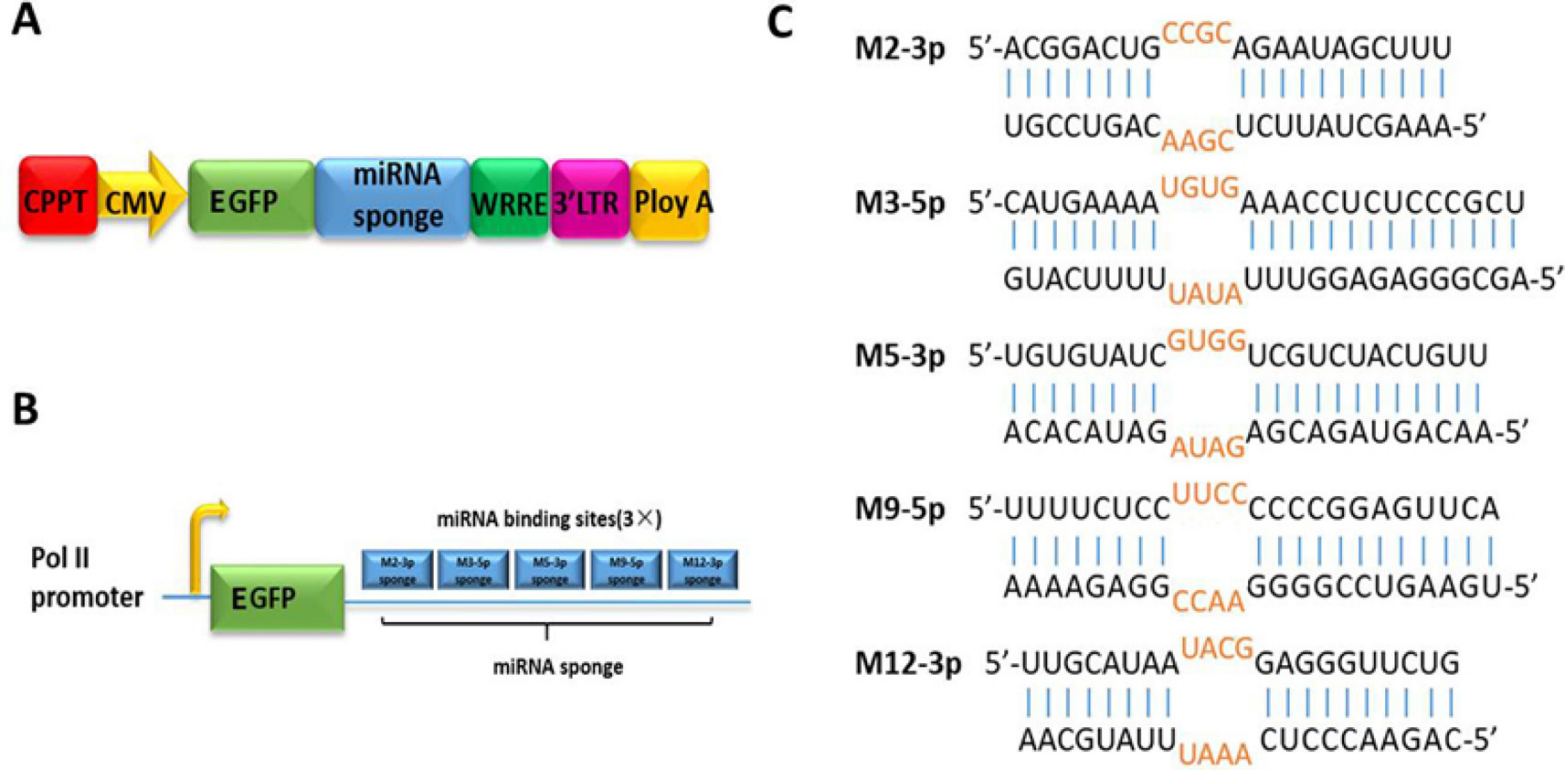
Stable expression of miRNA sponge for meq-cluster miRNAs by lentiviral vector (**A**) Lentiviral vectors were used to stably express miRNA sponge targeting meq-cluster miRNAs, in which CMV promoter drives the expression of EGFP. (**B**) Schematic diagram of miRNA sponge. (**C**) Sequences of the targeted miRNA and the corresponding miRNA sponge.

Five miRNAs of meq cluster have been selected as the sponge targets: M2-3p, M3-5p, M5-3p, M9-5p, and M12-3p. The lentiviral expression vector was used to express miRNA sponges, as shown Figure 1A.

As shown in Figure 1B, to improve the binding efficiency, the designed sponges have three binding sites for each miRNA target. In addition, in order to avoid the cleavage of Ago2 enzymes during RNA interference, bulge sequences were designed at the 9–12 nt position of the miRNA sequence to form mismatches to ensure the stability of miRNA sponges (Figure 1C).In the negative control group, the sponge sequence inserted in the lentiviral vector cannot bind to any target miRNA in the host (Supplementary Table 1).

### miRNA sponges degrade miRNAs of MDV-1 meq cluster

**Figure 2:**
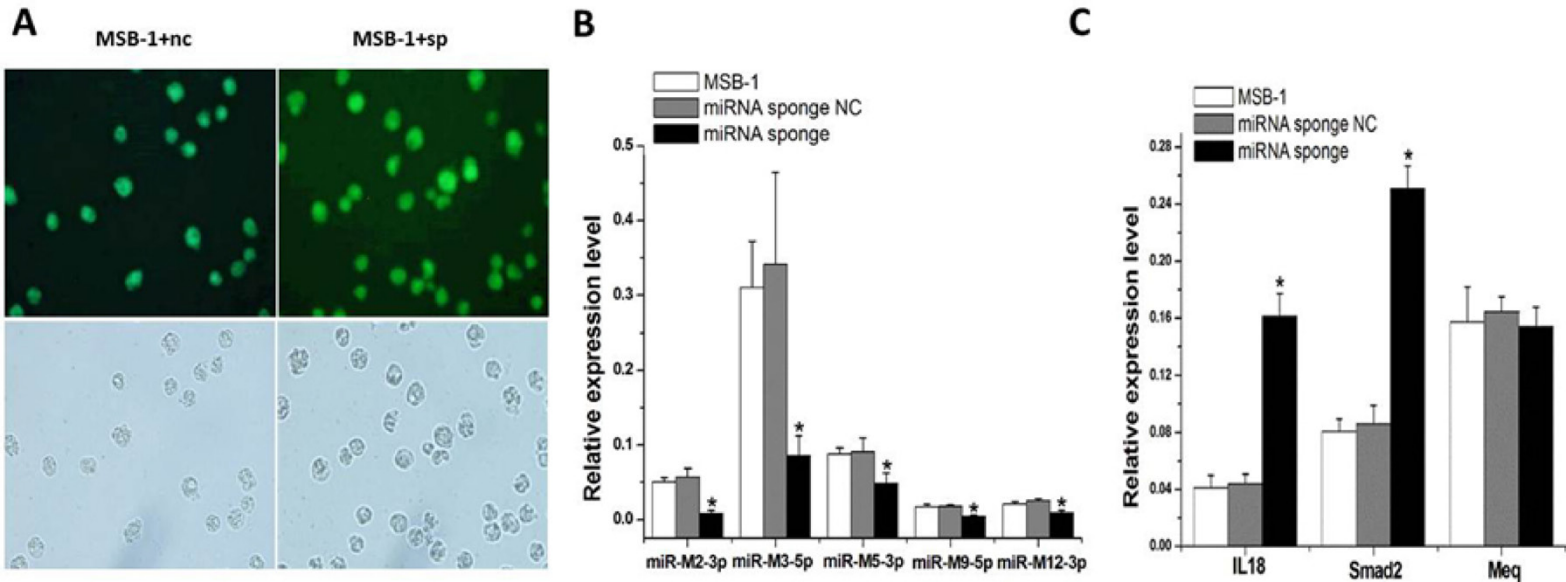
MiRNA sponge degraded MDV-1 meq miRNA cluster in MSB-1 cells (**A**) MSB-1 cells were infected with a miRNA sponge lentivirus or a miRNA sponge-NC expression virus (not bound to target miRNA). (**B**) The expression level of target miRNAs M2-3p, M3-5p, M5-3p, M9-5p, and M12-3p was measured in stable cell lines without infection, with miRNA sponge infection or miRNA sponge-NC infection. (**C**) mRNA levels of IL-18, smad2, and meq were measured in stable cell lines without infection, with miRNA sponge infection or miRNA sponge-NC infection. ^*^P < 0.05.

To demonstrate the effect of miRNA sponges on the expression of MDV-1 miRNAs, MSB-1 cell lines stably expressing miRNA sponges (Figure 2A) were constructed using lentivirus-mediated transfection (Supplementary Figures 1–3). We observed significant degradation of target miRNAs (*P* < 0.05) (Figure 2B). Sponges degrade miRNAs of meq cluster: miR-M2-3p, miR-M3-5p, miR-M5-3p, miR-M9-5p and miR-M12-3p. The mRNA expression of downstream genes IL-18 and smad2 were significantly higher than that of the control group, and the mRNA expression of MDV-1-specific gene meq did not change (Figure 2C).

### MDV-1 miRNA sponges inhibit proliferation of Marek’s disease virus-transformed MSB-1 cells

**Figure 3:**
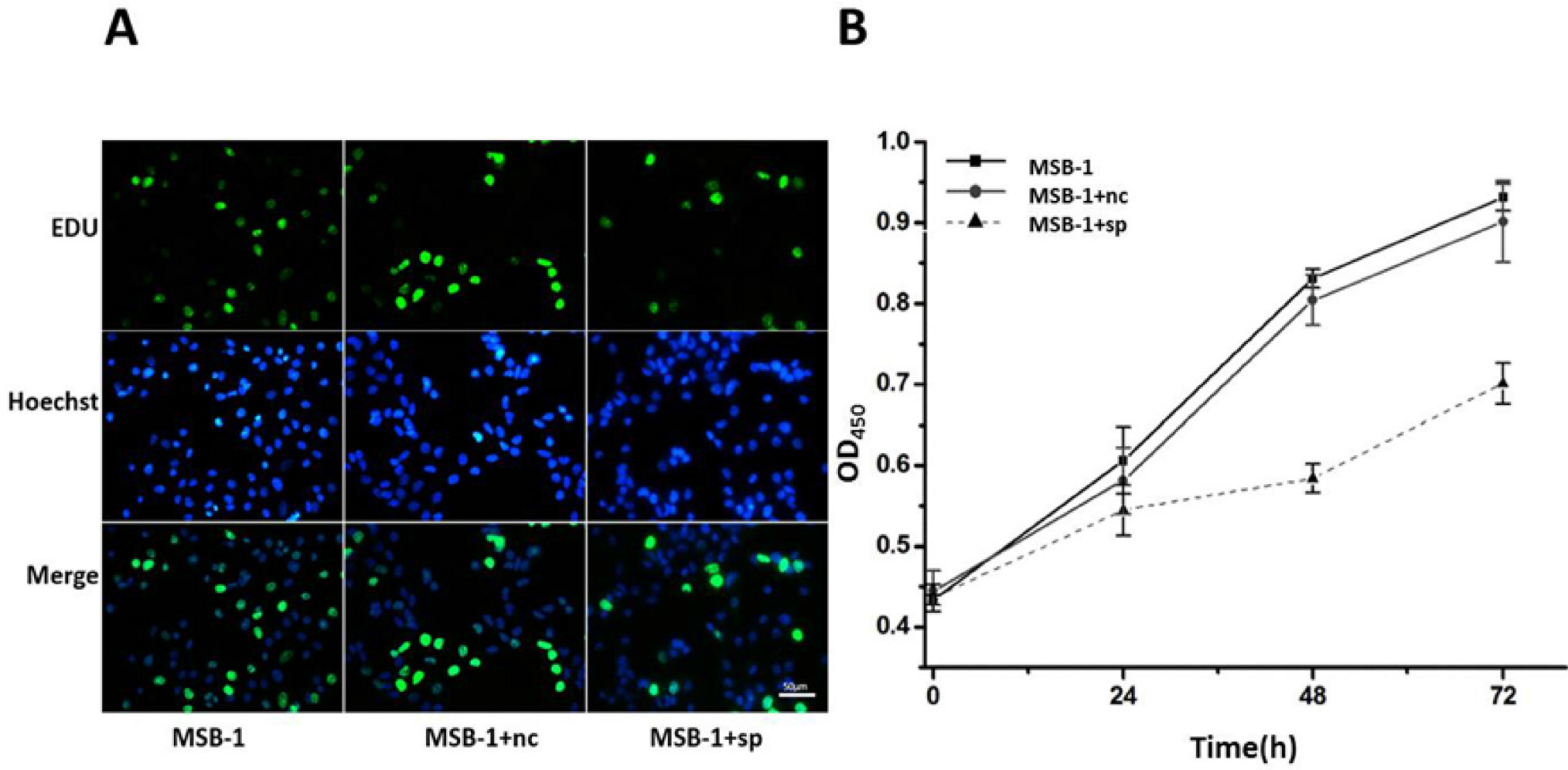
Effect of expression of MDV-1 miRNA sponge on proliferation of MSB-1 cells (**A**) miRNA sponge cells and control cells were incubated with EDU. After 24 h, EDU staining and DNA staining were used to observe the staining under fluorescence microscope. (**B**) The cells which were cultured in the incubator for 4 h were determined by OD value at 0 h. Then we determined OD at 24 h, 48 h, 72 h, respectively.

Because miRNA sponges can degrade pathogenic miRNAs expressed by MDV-1, we supposed that miRNA sponges can disrupt the physiological processes of Marek’s disease virus-transformed lymphoma cell line MSB-1, including proliferation, apoptosis and so on. To test this hypothesis, the staining of different cells after EDU labeling was examined. As shown in Figure 3A, compared with the control cells, the proliferation of MSB-1 cells expressing miRNA sponge was inhibited, compared to the control group. To further quantify the results of EDU staining, CCK8 reagent was added to 96-well plates, and the OD value measured at 0 h, 24 h, 48 h, 72 h could indirectly reflect the number of viable cells. Compared with control group, the proliferation rate of MSB-1 cells expressing miRNA sponges decreased obviously compared with that of control group (Figure 3B). Therefore, miRNA sponges can inhibit the proliferation of Marek’s disease virus-transformed tumor cells.

### MDV-1 miRNA sponges promote apoptosis of Marek’s disease virus-transformed MSB-1 cells

**Figure 4:**
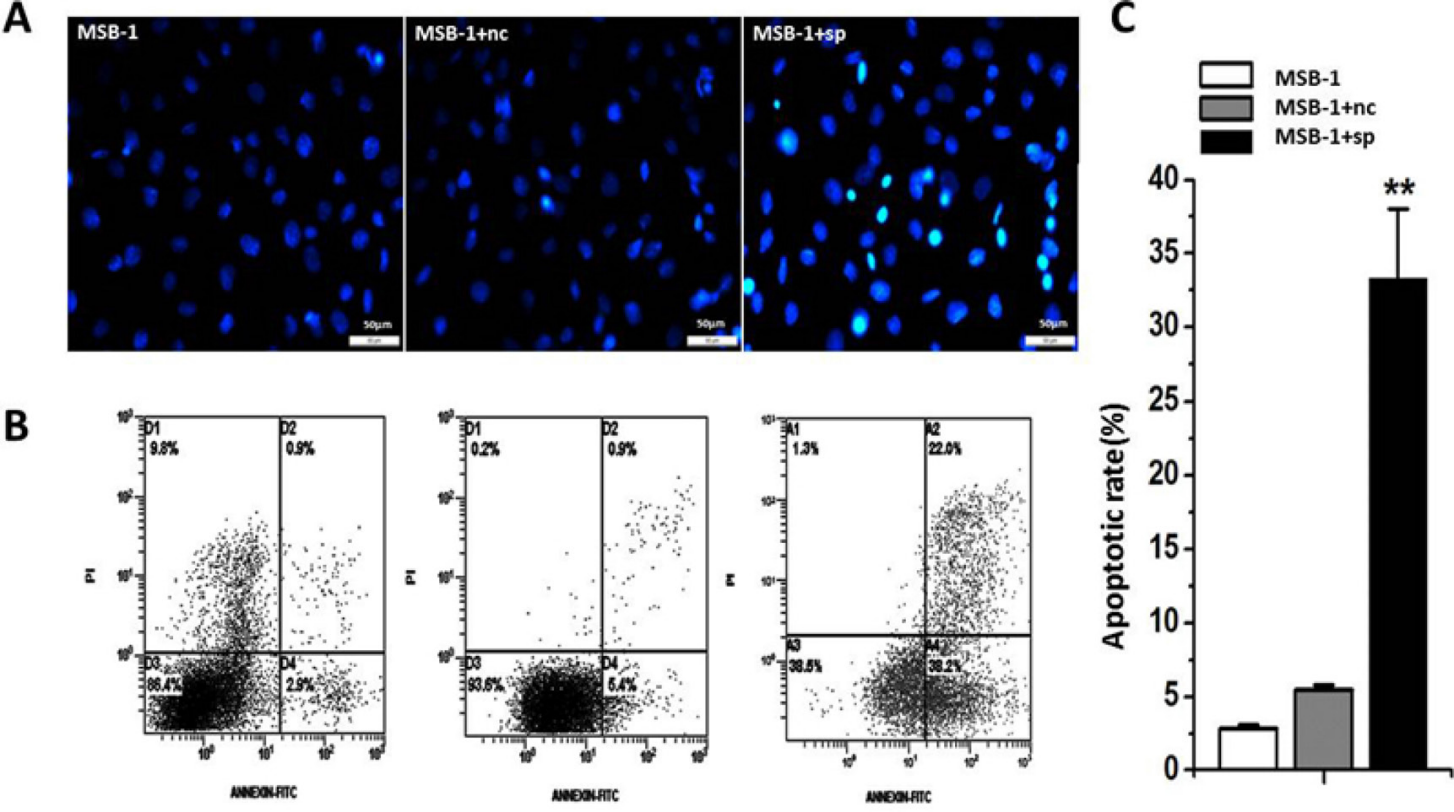
Effects of expression of MDV-1 miRNA sponge on apoptosis of MSB-1 cells (**A**) DAPI staining under the microscope visual chart. (**B**) After the Annexin V-FITC / PI double staining, the scatter plot was analyzed. (**C**) On the basis of the analysis of the fourth quadrant of the ratio of apoptosis. Data are means of at least three independent experiments. ^**^P < 0.01.

In the above experiments, we observed that the cell proliferation rate of the MSB-1 cells was decreased by miRNA sponges. This result may be caused by the effect of miRNA sponge on cell apoptosis. To test this, we used DAPI staining and Annxin V-FITC/PI double staining to detect the effect of miRNA sponge on apoptosis. Cells were stained with DAPI and photographed under inverted fluorescence microscope. The results showed that the percentage of apoptotic cells was significantly higher in MSB-1 cells expressing miRNA sponges than that of control and untreated group (Figure 4A). The flow cytometry was performed with Annexin V-FITC/PI double staining, and the early apoptotic rate of miRNA sponge group was 33.33%, which was much higher than that of control group and untreated group (5.47% vs 2.87%) (Figure 4B, 4C). In addition, we noted that there are some differences in the proportion of apoptosis between sponge control group and untreated group, indicating that lentivirus infection has a certain impact on the cells.

### MDV-1 miRNA sponges inhibit invasion of Marek’s disease virus-transformed MSB-1 cells

**Figure 5:**
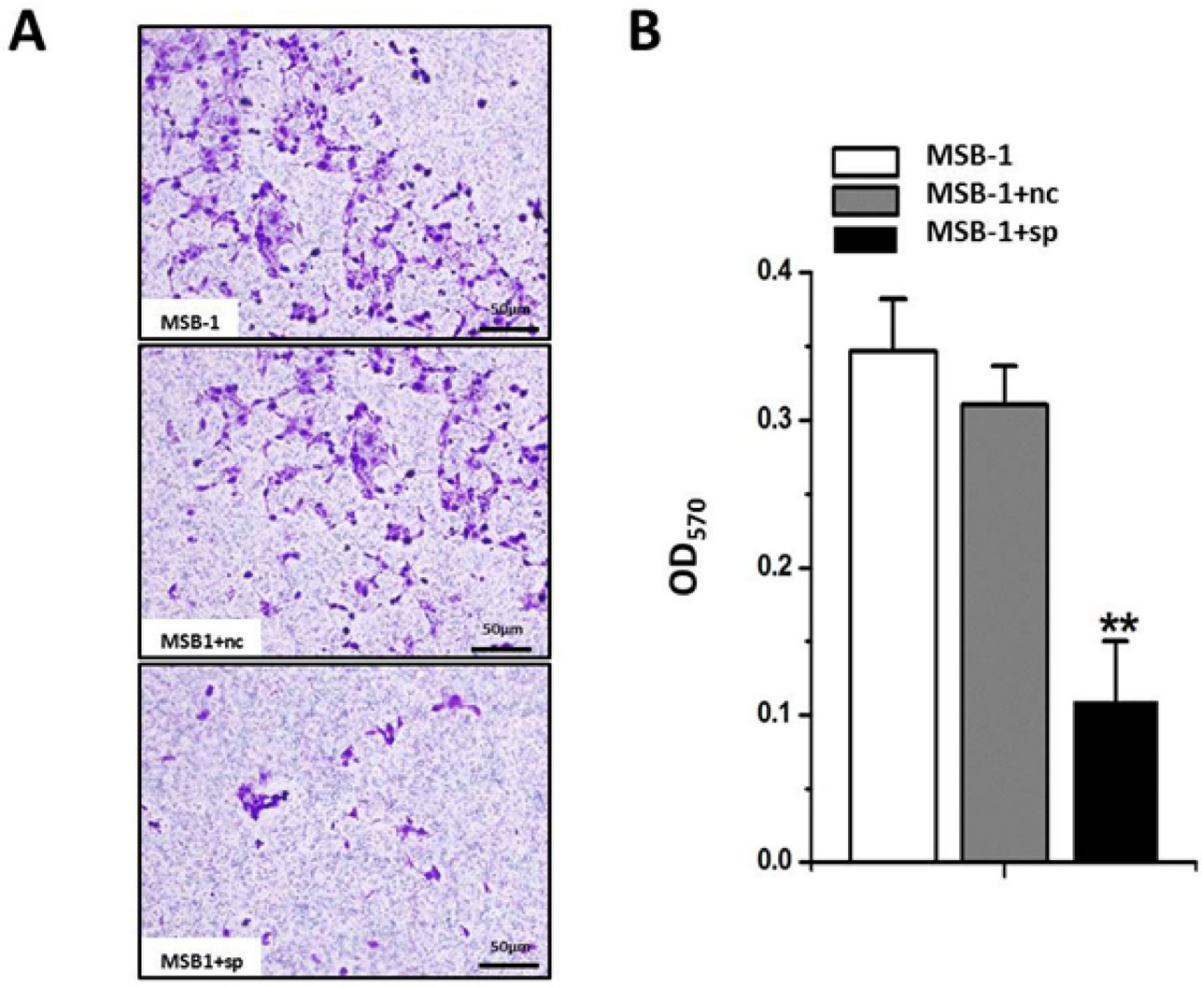
Effect of expression of MDV-1 miRNA sponge on invasion ability of MSB-1 cells (**A**) The visual diagram under the microscope after crystal violet staining. (**B**) After decolorization with 33% acetic acid, the OD value of the decolorization solution at 570 nm was examined. ^**^P < 0.01.

It is well known that the tumor incidence of the chicken host is significantly reduced in the presence of MDV-1 expressing no miRNA [26]. It is therefore speculated that pathogenic miRNA degradation may lead to a decrease in the invasion ability of the tumor cell line MSB-1. We performed the transwell assay, and the results show that (Figure 5A, 5B) invasion of MSB-1 cells expressing miRNA sponges has been decreased compared to the control group, which reveals that miRNA sponges can indeed inhibit the invasion of Marek’s disease virus-transformed MSB-1 cells.

### Effects of MDV-1 miRNA sponges on tumorigenicity of MSB-1 cells *in vitro*

**Figure 6:**
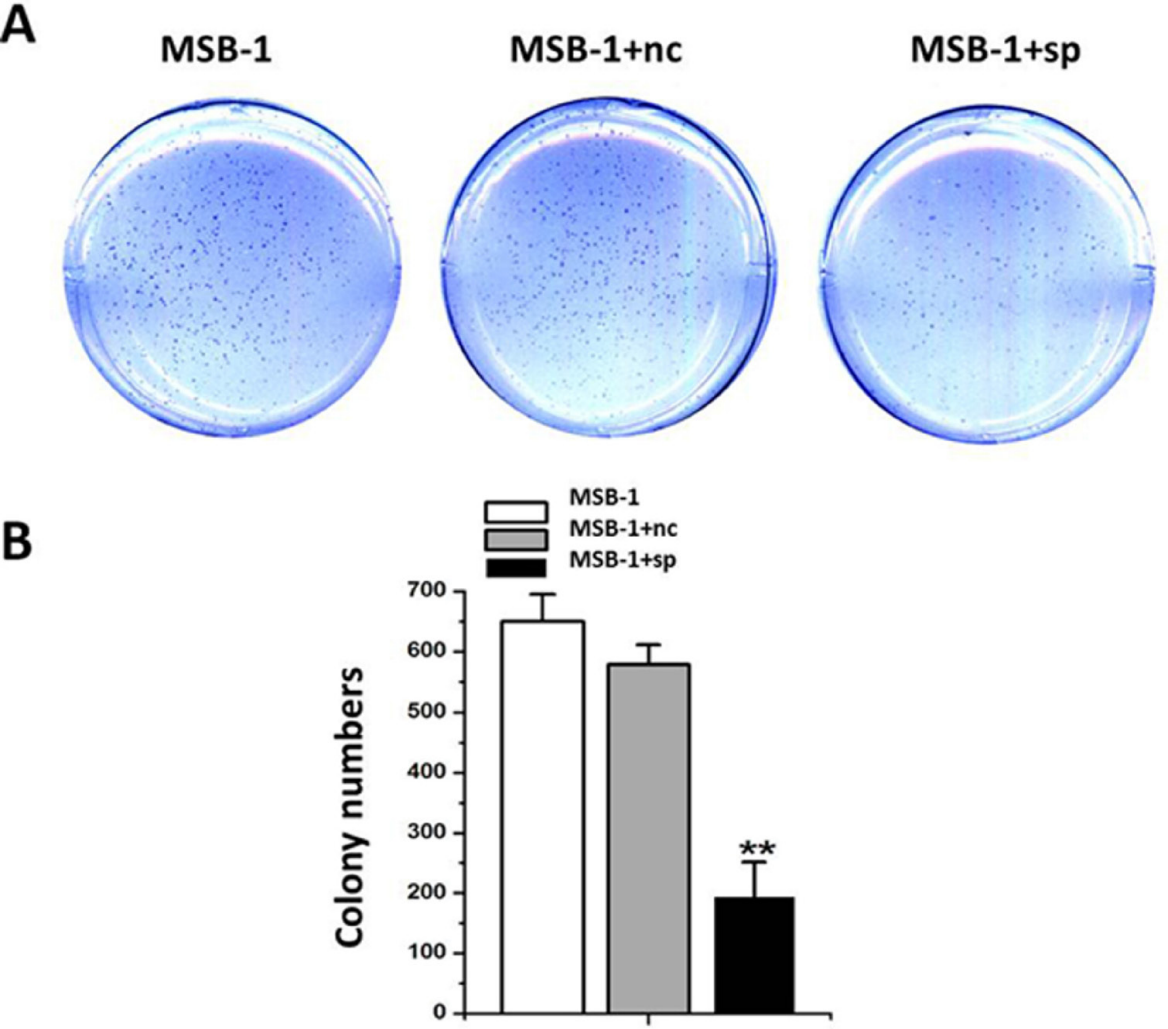
Effect of expression of MDV-1 miRNA sponge on tumorigenicity of MSB-1 cells *in vitro* (**A**) The cells treated with different treatments were cultured in a 37° C cell incubator for 12 days in a low melting point agar with an upper layer of 0.33% in 8,000 wells per well. The resulting clones were stained with crystal violet. (**B**) The clones formed were observed and counted (under eye view and microscope of 40 magnifications). ^**^P < 0.01.

To further demonstrate the effect of miRNA sponges on tumorigenicity of Marek’s disease virus, we performed the soft agar assay and the diluted crystal violet after staining was shown in Figure 6A. The number of clones formed in the miRNA sponge group was about 187.67, while the number of clones formed in the control group was greater than 550. The results were shown in Figure 6B. The number of clones in MSB-1 expressing miRNA sponges was about 1/3 of the control group, thus demonstrating that miRNA sponges inhibited the *in vitro* tumorigenesis of MSB-1 cells.

### Effects of MDV-1 miRNA sponges on growth rates of birds

To compare the pathogenicity of the sponge-treated MSB-1 cells with their non-treated cells, we examined the growth rates of inoculated birds. The experimental groups, each consisting of 25 one-day-old chickens, were separately inoculated with MSB-1 cells or sponge-treated cells by abdominal cavity inoculation while the negative control birds were inoculated with an equal volume of PBS solution.

**Figure 7:**
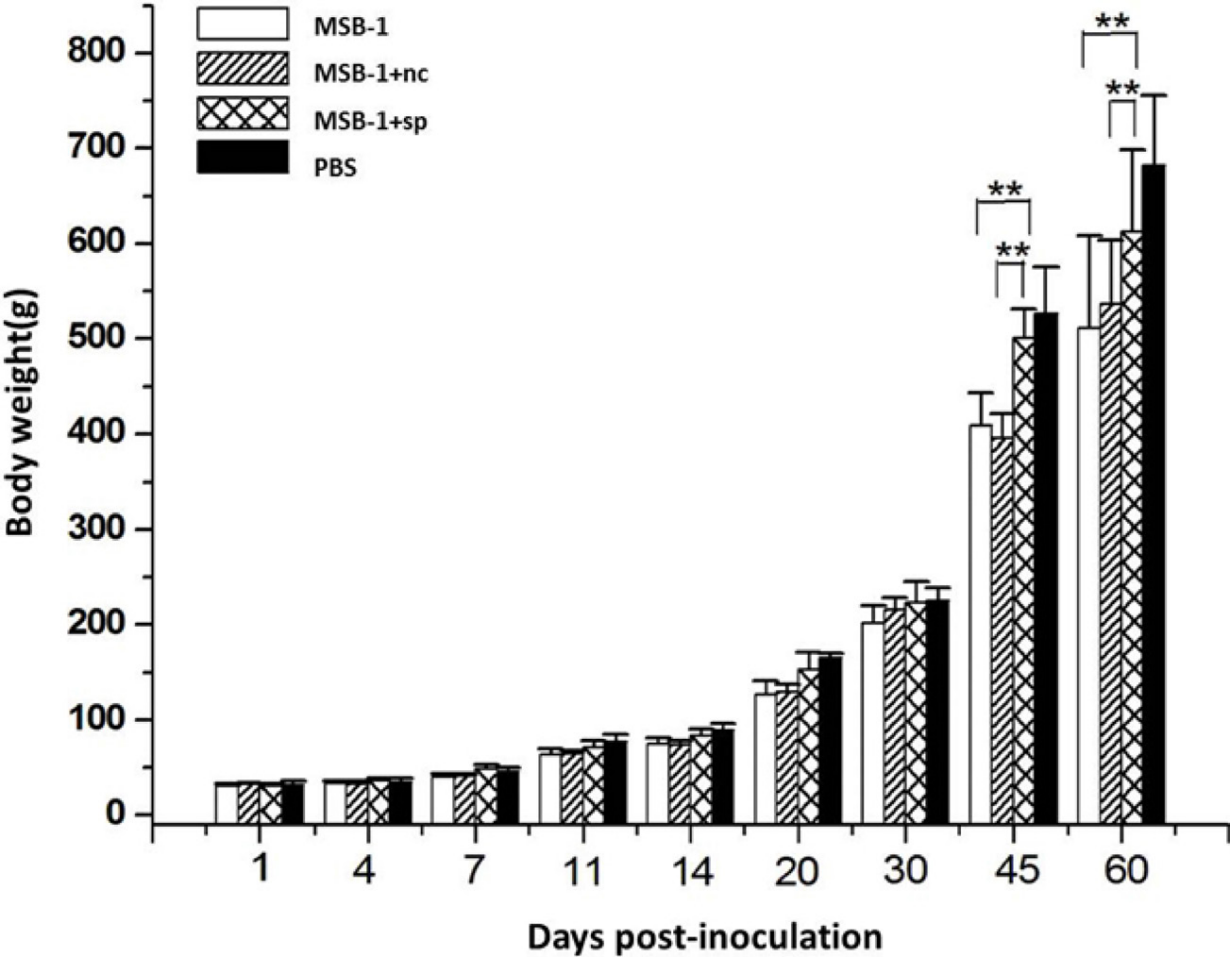
Comparisons of the growth rates of birds inoculated with distinct treated cells in 60 days ^**^P < 0.01.

As shown in Figure 7, no difference in weight of birds in all four groups was observed in the first month post-inoculation. After 30 days, the difference among four groups increased gradually. Both MSB-1 cells and MSB-1+nc cells strongly inhibited the growth rates of inoculated birds between 30 and 60 days. The birds inoculated with sponge-treated cells did not show this marked reduction in growth after 30 days p.i., although there is still a small reduction compared to chickens in PBS group.

### Pathogenicities of distinctly treated MSB-1 cells

**Figure 8:**
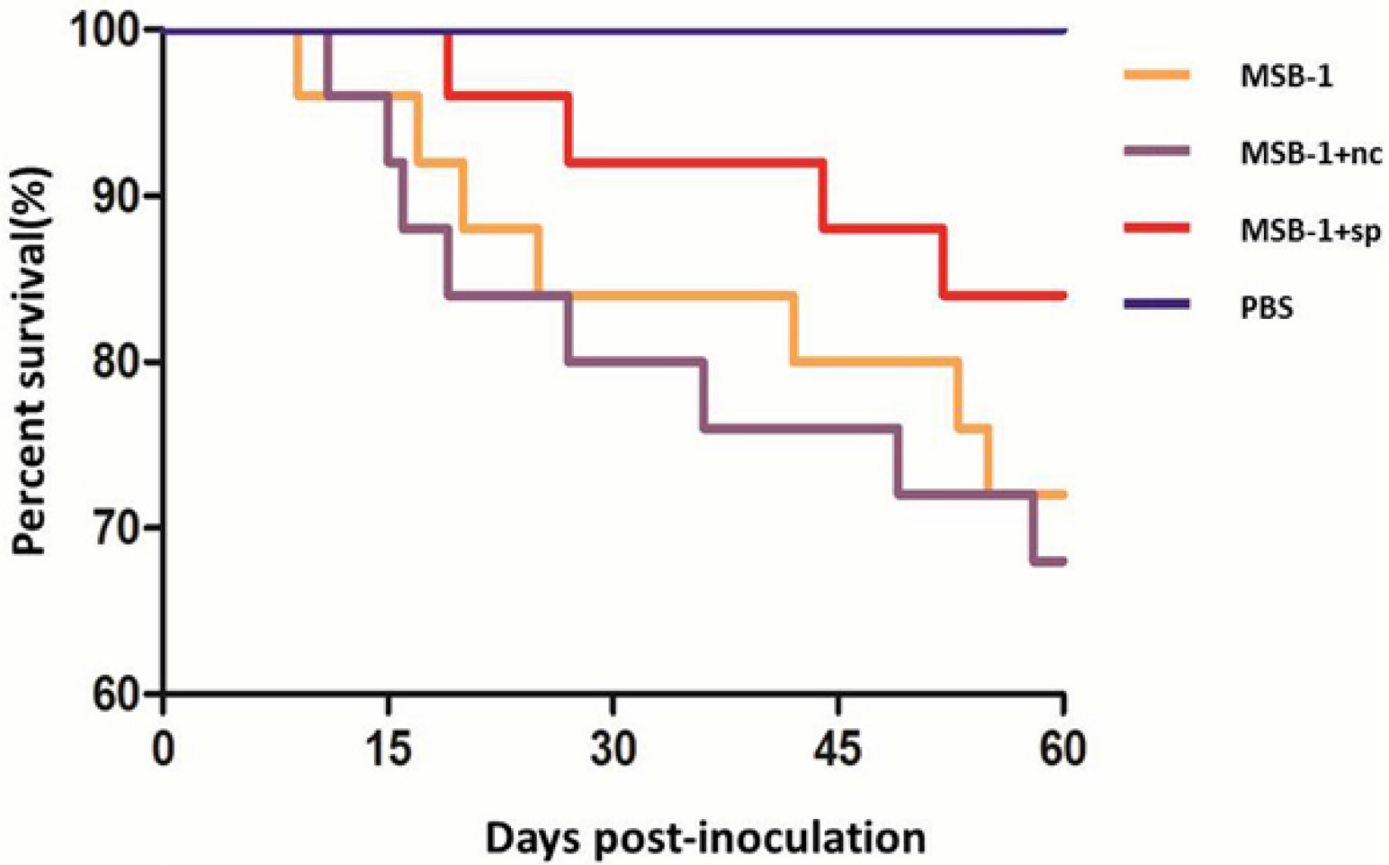
Survival curves of the birds inoculated with MSB-1 cells or its miRNA sponge-treated cells over the 60 day experimental time period

Post-inoculation, as demonstrated in Table 1, the chickens in MSB-1 group and MSB-1+nc group begun to show mortality before 14 days p.i., and mortality increased rapidly from 14 days p.i. to 30 days p.i. In contrast, birds inoculated with the sponge-treated cells did not show obvious signs of MD until about 14 days p.i. and exhibited low mortality throughout the experimental period. Thus, there were 72%, 68% and 84% of the birds inoculated with MSB-1 cells, MSB-1+nc cells and MSB-1+sp cells, respectively, remained alive at the end of the experimental period (Figure 8). In addition, by 60 days p.i., birds in MSB-1 group and MSB-1+nc group exhibited 12% and 8% tumor incidence (tumors mainly concentrated in spleens and livers); in contrast, birds inoculated with sponge-treated MSB-1 cells did not develop tumors, which was similar to PBS group. These results showed that compared to the MSB-1 cells, the pathogenicity and oncogenicity of the sponge-treated cells were strongly suppressed.

**Table 1:** Cumulative death and gross tumor occurrence in chickens inoculated with MSB-1 cells and its miRNA sponge-treated cells at different time points post inoculation

Time point	Category	MSB-1	MSB-1+nc	MSB-1+sp	PBS
1	Deaths	−	−	−	−
	Mortality	−	−	−	−
	Gross tumours	−	−	−	−
	Tumour incidence	−	−	−	−
7	Deaths	−	−	−	−
	Mortality	−	−	−	−
	Gross tumours	−	−	−	−
	Tumour incidence	−	−	−	−
14	Deaths	1	1	−	−
	Mortality	4%	4%	−	−
	Gross tumours	−	−	−	−
	Tumour incidence	−	−	−	−
21	Deaths	3	4	1	−
	Mortality	12%	16%	4%	−
	Gross tumours	−	−	−	−
	Tumour incidence	−	−	−	−
30	Deaths	4	5	2	−
	Mortality	16%	20%	8%	−
	Gross tumours	−	−	−	−
	Tumour incidence	−	−	−	−
45	Deaths	5	6	3	−
	Mortality	20%	24%	12%	−
	Gross tumours	−	−	−	−
	Tumour incidence	−	−	−	−
60	Deaths	7	8	4	−
	Mortality	28%	32%	16%	−
	Gross tumours	3	2	−	−
	Tumour incidence	12%	8%	−	−

Total of 10 birds in PBS group, and the other group was 25.

**–,** No death or gross tumors were observed at corresponding time points post-inoculation.

### Pathological lesions and tumorigenicity induced by sponge-intervened MDV-1

**Figure 9:**
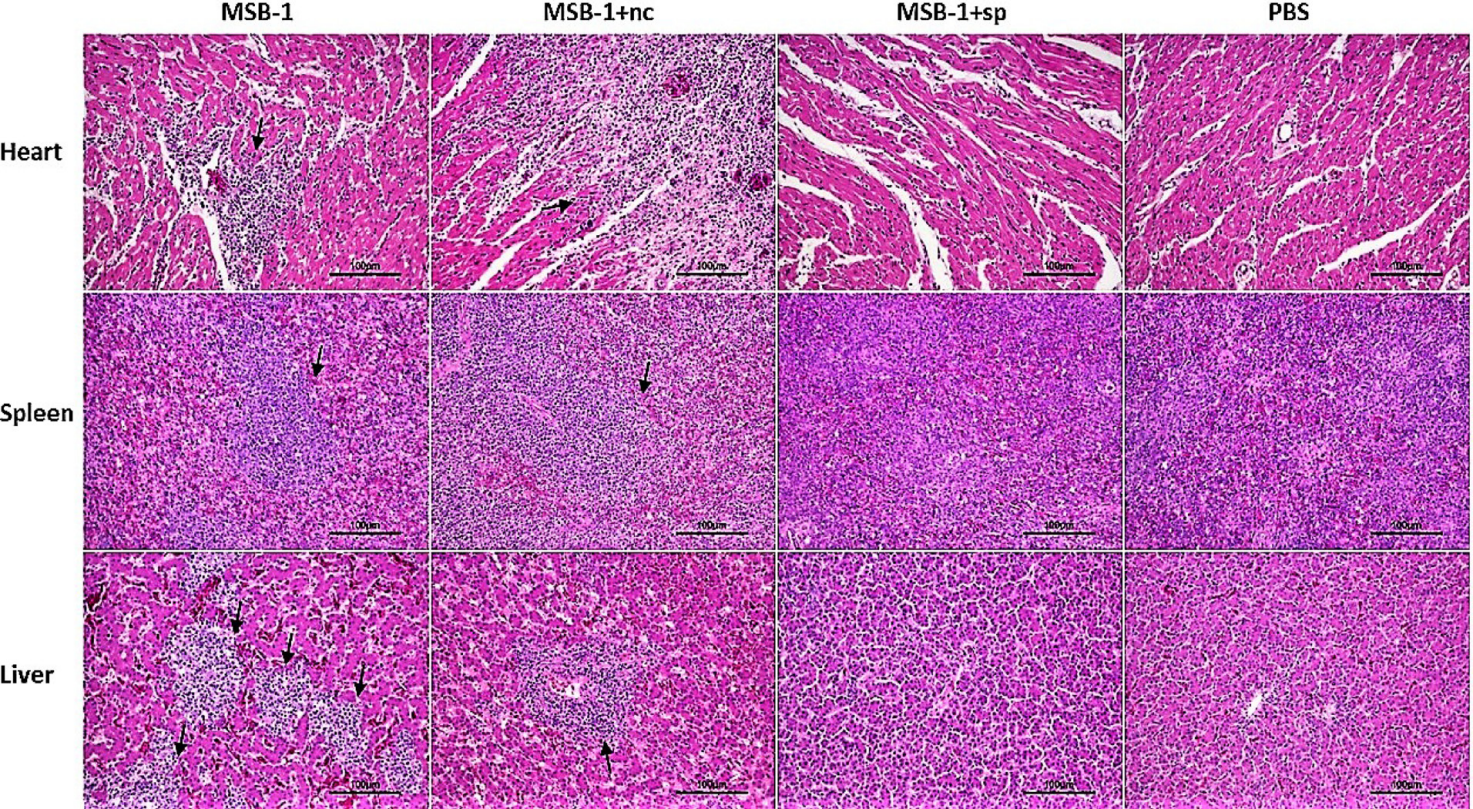
Pathological lesions and tumorigenesis in the hearts, spleens and livers of birds inoculated with MSB-1 cells or its miRNA sponge-treated cells at 60 days p.i. Tumor foci are shown by black arrows

To further examine the tumorigenicity in the distinct groups, all surviving birds from each experimental group were humanely euthanized at the end of the experimental period. Then we inspected and collected their pathological organs. As shown in Figure 9, tumor foci can be observed in the hearts, spleens and livers of MSB-1 group and MSB-1+nc group, whereas MSB-1+sp group showed no pathological lesion, which was similar to PBS group.

## DISCUSSION

MiRNA is a hotspot nowadays, and the relationship between tumors and viral miRNAs has become clear. For viruses, miRNA is an ideal tool for down-regulating tumor suppressor factors of host cells [20, 21]. Previous studies have shown that viral miRNAs are widely present in the herpes virus family, but are rarely found in other virus species [22, 23]. MDV belongs to the α-herpes virus subfamily. MDV-1-encoded miRNAs were first discovered in 2006, and 14 pre-miRNA sequences which produce 26 mature miRNAs have been identified. There are 3 miRNA clusters: meq clusters, Mid clusters and LAT clusters [24]. The meq miRNA cluster is considered to play a key role in promoting tumor development [15]. The expression of miRNAs from the meq gene cluster in the tumor caused by vv + MDV-1 infection was higher than that of the vvMDV-1 infection [25]. The expression of miRNAs in the tumor was significantly higher than that in the control group. In addition, the MDV1 miRNAs were highly expressed in lymphocytes and the expression of MDV and HVT miRNAs were also found in epidermal cells of the feather [26–28]. MDVl-miR-M3 can reduce the protein level of smad2 gene and achieve the anti-apoptotic phenotype in lesion cells by negative regulation of smad2 gene [29]. MDV1-miR-M2 and MDV1-miR-M9 can down-regulate mRNA of the host-encoded IL-18 gene. IL-18 is a proinflammatory factor that promotes the production of IFN-γ in T cells [30]. MDVl-miR-M4 suppresses host-encoded genes that were associated with tumor cell proliferation and apoptosis. However, it shares the same seed sequence with the host miR-155 [15]. In order to avoid breaking the normal signaling pathway in the host, we have selected five pathogenic miRNAs except miR-M4 belong to the MDV-1 meq cluster: miR-M2, miR-M3, MiR-M5, miR-M9 and miR-M12.

At present, the main methods for detecting miRNA expression levels include miRNA cloning, northern-blotting and real-time quantitative PCR. Yao *et al.* used real-time quantitative PCR to detect miRNA expression in MSB-1 cells and found that various MDV-1 miRNAs including MDV-1 miR-M2-3p, miR-M3-5p, miR-M5-3p, miR-M9-5p, miR-M5-5p, miR-M5-3p, miR-M5-3p, miR-M5-5p, miR-M5-5p and MiR-M12-3p were stably expressed in MSB-1 [24].

In order to efficiently and steadily inhibit the target miRNAs, lentiviral vector was used to express the miRNA sponges. In this study, it was confirmed that miRNA sponges could inhibit the proliferation, invasion and tumorigenesis of MSB-1 cells *in vitro* and promote cell apoptosis by binding to target miRNA molecules of MDV-1 meq cluster in host cells. Furthermore, animal experiments showed that miRNA sponge could inhibit the growth of MSB-1 cells, effectively alleviate the inhibition of MSB-1 cells on growth of SPF chickens, improve the survival rate of chickens and reduce the rate of tumor formation. This study confirms the effective role of miRNA sponges in degrading MDV-1 specific miRNAs and interfering with its pathogenicity and tumorigenicity. It provides a potentially effective anti-tumor small molecule for the cancers caused by herpes virus, including Marek virus.

## MATERIALS AND METHODS

### Construction of miRNA sponge vector

We used pUC57-Kan-MCS vector (Life technologies)as a backbone. Firstly, 5 miRNAs in meq-cluster were chosen as our targets: mdv1-miR-M2-3p, -miR-M3-5p, -miR-M5-3p, -miR-M9-5p and -miR-M12-3p. Then, we designed and synthesized their sponge sequences and control sequences. To enhance their binding activity, each sponge sequence corresponding to its targeted miRNA repeated three times. In these sequences, –CUUC- served as a linker. After that, synthesized sequences were digested by Esp 3I site and inserted into pHS-BMR-LW001 vector. Beijing Syngentech Company taken this job, subsequently we verified it by Hind III digestion and sequence align.

### Cell lines and cell culture

MSB1 is a CD4+T cell line derived from a spleen lymphoma induced by the BC-1 strain of MDV-1. It is recognized as a brilliant model for lymphmas research. Cells kindly provided by Prof. Aijian Qin of Yangzhou University were cultured in RPMI-1640 medium(Gibco) supplemented with 10% fetal bovine serum (FBS, Gibco) and 10% TPB(Sigma) under 5% CO_2_ at 38.5***°*** C.

For the lentivirus production, human embryonic kidney(HEK)293T cells were cultured in DMEM(Gibco) supplemented with 10% FBS(Gibco), 100 U/ml penicillin, 100 g/ml streptomycin, sodium pyruvate and L-glutamine at 37° C with 5% CO_2_.

### Chickens

Experiments were conducted in specific-pathogen-free (SPF) white Leghorn chickens Suplied by Guangdong Wens foodstuff Co. Ltd. that were maintained in isolators with filtered air under positive pressure in an SPF animal facility.

### Lentivirus production and cell transduction

Lentivirus production:pHS-AMR-LW001 vector and pHS-AMR-LW002 vector were respectively cotransfected with psPAX2 and pMD2.G into HEK293T cells using PEI-MAX (Ploysciences). Viruses were harvested at 72 h after transfection and viral titers were determined.

Cell transduction: to generate inducible sponge-expressed cell line, we infected MSB-1 cells with distinct packaged lentivirus. After infection, we selected these cellswith 150 ug/ml Zeocin (Sigma) for 7 days. The resulting cells were tested for sponge induction after flow sorting and checked the expression of EGFP under fluorescence microscopy (Olympus).

### RNA extraction and quantitative real-time PCR

After transduction and flow sorting, 1 × 10^6^ cells were collected and the total RNA of different groups were extracted using the Trizol reagent (Invitrogen, USA) according to the manufacturer’s protocol. Synthesis of cDNA was performed by using SuperScript III^®^ (Invitrogen) according to the manufacturer’s instructions. For quantitative assessment of targeted miRNA, TRIzol-isolated RNAs were reverse transcribed by miScript ll RT Kit and measured by miScript SYBR Green PCR Kit in LightCycler 480 II. Cycle threshold (Ct) values of the analyzed miRNAs were normalized to Ct values obtained for the noncoding, small nuclear RNA molecule U6. Data were expressed as fold change versus control. Quantitative real-time PCR was performed using the ABI PRISM 7000 Fluorescent Quantitative PCR System (Applied Biosystems, Foster City, CA, USA) according to the manufacturer’s instructions. Expression fold changes were calculated using 2^−ΔΔCt^ methods.

### Cell proliferation assay

EdU incorporation assay: To evaluate the proliferation of the distinct treated cells, 50 μM 5-ethynyl-2′-deoxyuridine (EdU; Ribobio, China) was added to the medium for 4 h. To determine the incorporation of EdU, the cells were fixed with 4% paraformaldehyde for 30 min at room temperature, and immunostained (red) using a standard protocol; in addition, Hoechst stain (blue) was used to visualize the nucleus. EdU incorporation was viewed and captured.

CCK 8 assay: Cells were incubated in 10% CCK-8 (Dojindo; Kumamoto, Japan) that was diluted in normal culture medium at 37° C until the visual color conversion occurred. Proliferation rates were determined at 0, 24, 48, and 72 hours after transfection.

### Cell apoptosis assay

Apoptotic morphology: for morphological examination, distinct treated MSB-1 cells were stained with 4′,6′-diamidino-2-phenylindole (DAPI; Sigma-Aldrich), and those with fragmented or condensed nuclei in deep staining were counted as apoptotic cells.

Annexin V-fluorescein isothiocyanate/propidium iodide (FITC/PI) assay: for the treated cells, an annexin V-FITC/PI assay (BD Biosciences Pharmingen) was performed according to the manufacturer’s protocol; after staining, cells were analyzed by a FACSCalibur instrument (Becton Dickinson, San Jose, CA). Experiments were repeated at least three times in duplicates.

### Cell invasion assay

For the invasion assay, the inserts were precoated with extracellular Matrigel (BD Biosciences, USA) diluted with RPMI1640 (Gibco) to a certain percentage and incubated at 37° C overnight. Then, different sponge-treated MSB-1 cells were suspended in non-FBS RPMI1640 and seeded in the upper chambers, and RPMI1640 containing 20% FBS was added to the lower chamber. After incubation at 37° C for 26 h , the cells were fixed and stained with 0.4 mg/mL Crystal Violet solution.

### Soft agar colony forming assay

Different sponge-treated MSB-1 cells were plated in 1.33% low melting point agar was prepared and mixed with RPMI1640 containing 20% FBS to make 0.33% and 0.66% agar at 45° C. 1 mL 0.66% agar was added into the bottom of the 6-well plate. 8 × 10^3^ cells/well was mixed with 1 ml 0.33% agar and added on the top of 0.66% agar. Cells were allowed to grow for 12 days. Cells were stained with 0.4 mg/mL Crystal Violet solution on the plates and counted.

### Animal experiments

The animal experiments with birds were performed according to the local protocols of the Ethical and Animal Welfare Committee of Key Laboratory of Animal Immunology of the Ministry of Agriculture of China. In experimental groups, each of 25 one-day-old white Leghorn SPF chickens was separately inoculated with distict treated MSB-1 cells by abdominalcavity inoculation. We inspected their clinical symptoms, and meanwhile recorded their death numbers and gross tumors in each group. At the end of 60 days, all surviving birds were humanely euthanized and their organs were examined for lesions at necropsy.

### Evaluation of effects of sponge-treated MSB-1 cells on growth rates

In order to evaluate the effects of sponge-treated MSB-1 cells on the birds’ growth rates, we weighed all birds from each group and recorded at 1, 4, 7, 10, 14, 21, 30, 45 and 60 days p.i.

### Pathological lesions and tumor induction by MDV-1

Pathological lesions and tumour induction by MDV-1. At 60 days p.i., all surviving birds from each experimental group were humanely euthanized. Hearts, spleens and livers were collected, fixed in 10% formalin and processed for embedding in paraffin. Three micrometre thick sections of the tissues were prepared and stained with haematoxylin and eosin (HE).

### Statistical analyses

All experimental data from three independent experiments were analyzed by Student’s *t*-test or ANOVA and *P* < 0.05 was considered statistically significant. All statistical tests were conducted by SPSS version 19.0 software (SPSS Inc.Chicago, IL, USA).

## SUPPLEMENTARY MATERIALS FIGURES AND TABLE


