# Supplementary material for: Design of miRNA sponges for MDV-1 as a therapeutic strategy against lymphomas
Source: Oncotarget. 2017 Dec 17;9(3):3842–52. doi: 10.18632/oncotarget.23379 (PMC5790504; doi:10.18632/oncotarget.23379)
Supplement: Supplementary file 1 [file oncotarget-09-3842-s001.pdf]

Design of miRNA sponges for MDV-1 as a therapeutic strategy against lymphomas

SUPPLEMENTARY MATERIALS

Electrophoresis and sequencing results proved that the vector has been constructed successfully

miRNA sponges and the negative control sequences were synthesized and then inserted into the EGFP 3′UTR of the lentiviral vector pHS-BMR-LW001. After construction, the vector was digested by endonuclease Hind III (Figure 1A) and sequenced (Figure 1B). Electrophoretic bands, and sequencing results are consistent with expectations.

Lentivirus packaging

The lentiviral vector miRNA sponge and its control vectors were transfected into the well-behaved 293T cells, and Figure 2 shows the white and fluorescent images of the cells taken before and after transfection.

In order to detect the titer of lentivirus after concentration, the concentrated lentivirus was diluted and added to different wells of the 96-well plate. Each group was repeated 3 times. After 72 h, the cells were observed under fluorescence microscope (Figure 3), and lentivirus titers were calculated according to the formula: Titer (TU/mL) = (a + 10 × b) × 1000/2/a. In this formula, a, b is the number of fluorescent cells recorded in the last two wells that can be observed with fluorescence (Table 1). As calculated by the above formula: titer of lentivirus expressing miRNA sponge (TU/mL) = 2.45×10^6; titer of lentivirus expressing miRNA control sponge (TU/mL) = 1.12×10^6.

Supplementary Table 1: The number of fluorescent cells can be observed in the last 2 wells

Category	Volume (μL)	1	2	3	Average
miRNA sponge	10^{-2}	24	32	31	29
miRNA sponge	10^{-3}	2	2	2	2
miRNA sponge-NC	10^{-2}	15	14	18	15.67
miRNA sponge-NC	10^{-3}	1	0	1	0.67

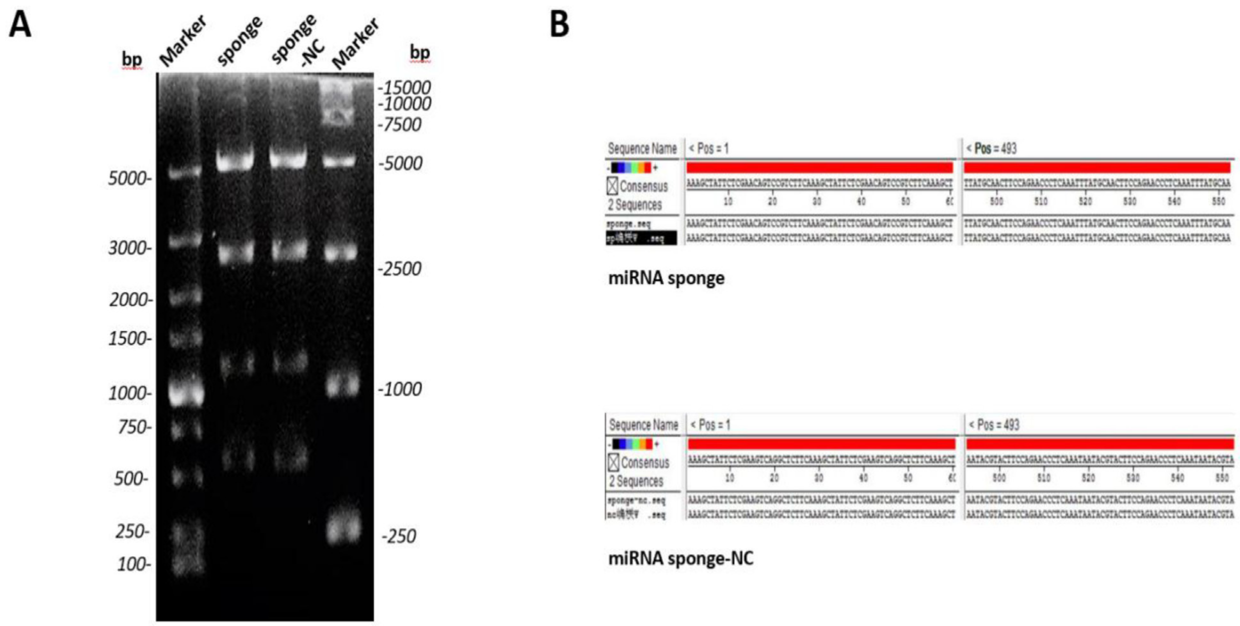

Supplementary Figure 1: Verification of lentiviral vector miRNA sponge and miRNA sponge-NC. (**A**) Eectrophoresis of lentiviral vector miRNA sponge and miRNA sponge-NC vector after *Hind* III digestion (L:Takara DL5000 marker,R:Takara DL15000 marker). (**B**) Sequencing results of lentiviral vector expressing miRNA sponge and miRNA sponge-NC and the comparison of targeted sequences.

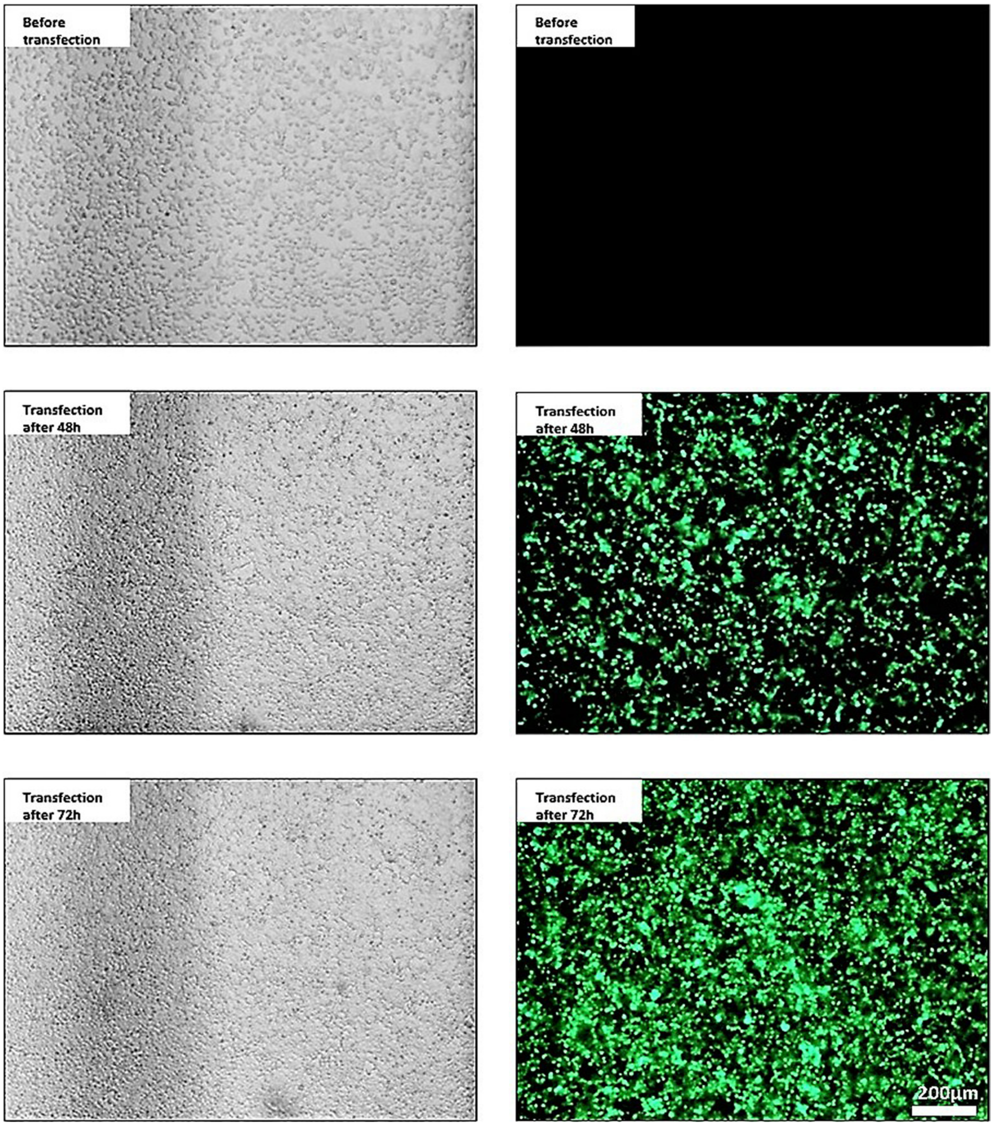

Supplementary Figure 2: Packaging of lentivirus miRNA sponge.

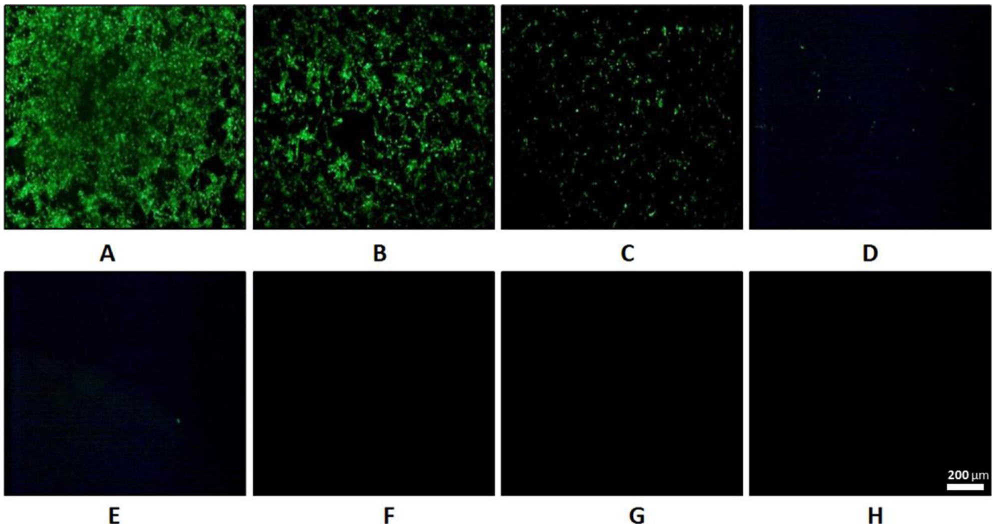

Supplementary Figure 3: Detection of miRNA sponge expressed lentivirus titer by fluorescence titration. The fluorescence of each well in 72 h after adding different volumes (10, 10^0, 10^{-1}, 10^{-2}, 10^{-3}, 10^{-4}, 10^{-5}, 10^{-6} μL) of concentrated lentivirus to the 96-well plates, in which 293T cells was incubated. Different volume is corresponded to (**A–H**).
